# Green Precursors and Soft Templating for Printing Porous Carbon‐Based Micro‐supercapacitors

**DOI:** 10.1002/chem.202003124

**Published:** 2020-12-07

**Authors:** Stefanie Lochmann, Susann Kintzel, Yannik Bräuniger, Thomas Otto, En Zhang, Julia Grothe, Stefan Kaskel

**Affiliations:** ^1^ Chemistry and Food Chemistry Inorganic Chemistry I Bergstraße 66 01159 Dresden Germany

**Keywords:** micro-supercapacitors, nanoimprint lithography, quasi-solid-state, soft lithography, soft templating

## Abstract

A combination of soft lithographic printing and soft templating has been used to fabricate high‐resolution interdigitated micro‐supercapacitors (MSC). Surfactant‐assisted self‐assembly produces high surface area ordered mesoporous carbons (490 m^2^ g^−1^). For the first time, such precursors have been printed by nano‐imprint lithography as microdevices with a line width of only 250 nm and a spacing of only 1 μm. The devices are crack‐free with low specific resistance (1.2×10^−5^ Ωm) and show good device capacitance up to 0.21 F cm^−3^.

## Graphical abstract

**A fine line**: A combination of soft lithographic methods and soft templating was used to fabricate high‐resolution micro‐supercapacitors (MSC). Surfactant‐assisted self‐assembly produces high surface area ordered mesoporous carbons. For the first time, such precursors can be printed by nano‐imprint lithography as microdevices with a line width of only 250 nm and a spacing of only 1 μm.

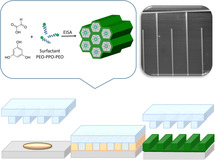

## Introduction

An increasing demand for flexible and portable energy‐storage systems powering micro‐electromechanical systems (MEMS), implantable medical devices, and the internet of things requires extensive developments in on‐chip energy storage systems.[^1^, ^2^] In the field of rechargeable micro‐power devices micro‐batteries are being extensively investigated.^[3]^ Their lack of cycling stability and low power densities can be overcome by using electrochemical double‐layer capacitors (EDLC).[^4^, ^5^, ^6^] Their energy‐storage capacity is based on the electrostatic adsorption of electrolyte ions on the inner surface of highly porous carbons. This physical ion‐storage mechanism without faradaic reactions enables fast charge–discharge rates and remarkable cycling stabilities over millions of cycles. Hence, systems providing high power densities are commercialized.[^7^, ^8^, ^9^]

A key feature of micro‐supercapacitors is their two‐dimensional geometrical design, which is based on interdigitated structures integrated on a flat surface, rendering such systems ideal for microelectronic device integration. These structures enable an increase in surface area and foster a good electrode accessibility and shorter diffusion pathways. With the fixed electrode setup, the use of separators may be dispensed with; this further facilitates the ion transport.[^2^, ^4^] Direct structuring and deposition methods enable binder‐free electrodes, further improving the performance.[^1^, ^10^]

Solvent‐assisted nanoimprint lithography (SA‐NIL) is an elegant method to directly create an interdigitated pattern on a flat surface. Micro‐ and nanostructured electrodes can be fabricated by using this low‐cost, high‐throughput method.^[11]^ NIL typically achieves higher resolution (down to nanoscale) than simple inkjet printing or laser micro‐structuring. Moreover, the liquid precursor will not cause particle contamination, as subtractive laser structuring is accompanied by massive dusting causing shunts.

Soft lithography is based on patterning a precursor with an elastomeric stamp.[^12^, ^13^] Scalability has been demonstrated by integration into roll‐to‐roll processes.[^14^, ^15^] In the case of SA‐NIL, a liquid and curable precursor is customized for printing, hardening and thermal transformation into crack‐free line patterns consisting of a high surface area material with defined porosity.

In the field of MSCs, a wide range of materials and various structuring approaches have been investigated in recent years. Appropriate electrode materials supplying highly active surface areas and good electrical conductivities are activated carbons,[^16^, ^17^, ^18^, ^19^] conducting polymers,^[20]^ MXenes,[^21^, ^22^] or metal oxides (RuO_2_, MnO_2_).^[23]^ The use of various composite materials is also widely established.^[24]^ In the area of porous carbons several materials such as carbide‐derived carbon (CDC),[^25^, ^26^, ^27^, ^28^] graphene,[^29^, ^30^] or CNTs[^31^, ^32^, ^33^] with high specific surface areas (SSAs; >1000 m^2^ g^−1^) have been investigated for MSCs in the past.^[34]^

Customized liquid carbon precursors such as phenolic resins made of phenol or resorcinol in combination with formaldehyde fulfill the demands for SA‐NIL.[^35^, ^36^, ^37^] However, carcinogenicity and low environmental compatibility are disadvantageous for sustainable production and safe workspaces. From this point of view, Ghimbeu et al. have presented promising precursors based on so called “green resoles” that contain phloroglucinol and glyoxylic acid as monomers and react in a catalyst‐free polymerization.^[38]^

In the following, we report the development and excellent performance of “green resoles” combined with soft templating for the SA‐NIL processes by using an UV‐assisted evaporation induced self‐assembly (UV‐EISA) for micelle assembly that enables one‐step curing of the precursor during the NIL‐process.

## Results and Discussion

**Figure 1:**
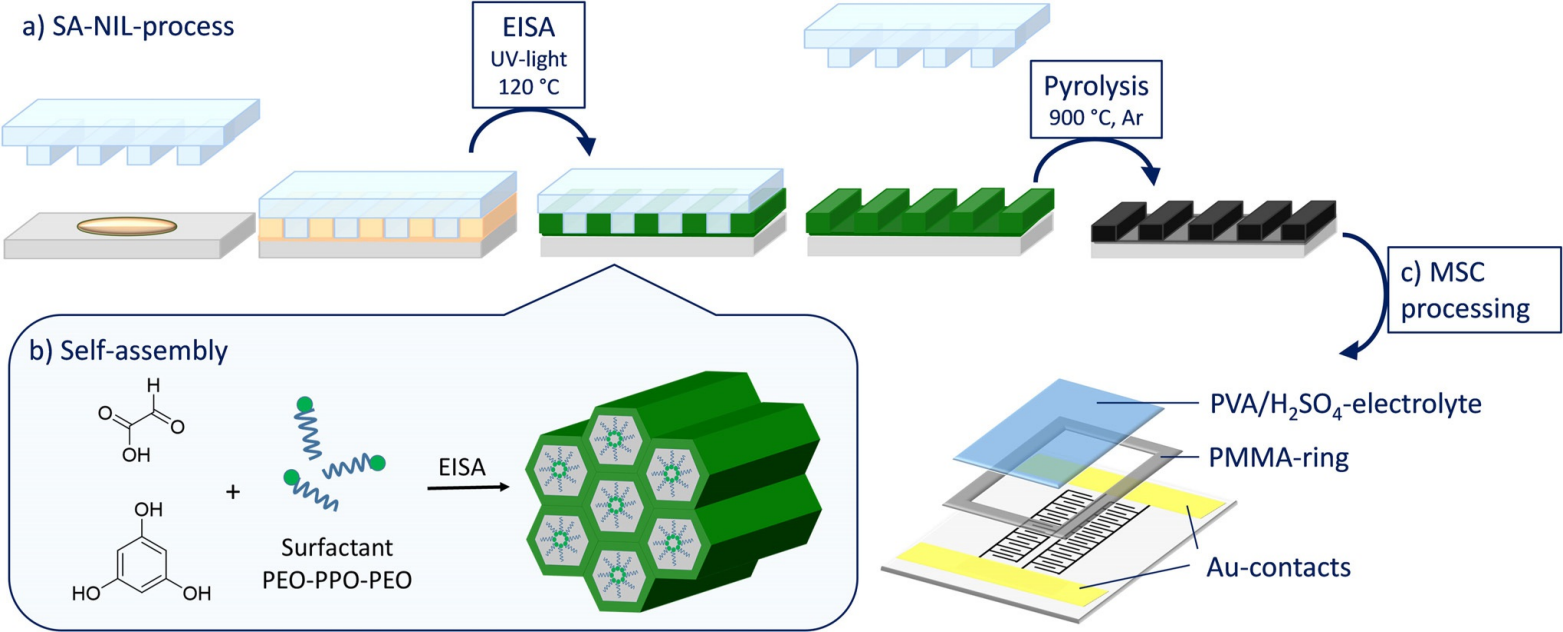
a) Schematic SA‐NIL procedure with b) in situ evaporation induced self‐assembly of the precursor solution and c) further MSC processing steps.

The SA‐NIL process is ideal to produce in‐plane micro‐supercapacitors with interdigitated geometry.^[12]^ Precursor development is crucial to enhance the capacitance or tailor the frequency response. The precursor needs to fulfill a list of requirements with respect to patterning, resolution, structural stability, and the resulting porous carbon electrical conductivity, pore size distribution and surface area. The requirements for the application of a liquid precursor in the SA‐NIL‐process are, that the precursor completely cures under thermal or UV treatment and results in homogeneous structures with good dimensional stability. In this work we customize the green carbon precursor based on phloroglucinol and glyoxylic acid. The phenolic resin is suitable for the pattering process because of its fast polymerization during temperature and UV treatment. Furthermore, the addition of a surfactant like Pluronic F127 enables soft templating in order to induce a defined porosity.^[39]^ In the following we compare a non‐templated and a soft templated carbon precursor for SA‐NIL applications (Figure 1). The precursors are printed in two different interdigitated patterns with line widths of 250 (IDE250) and 500 nm (IDE500).

### Carbon precursor and thin film characteristics

**Figure 2:**
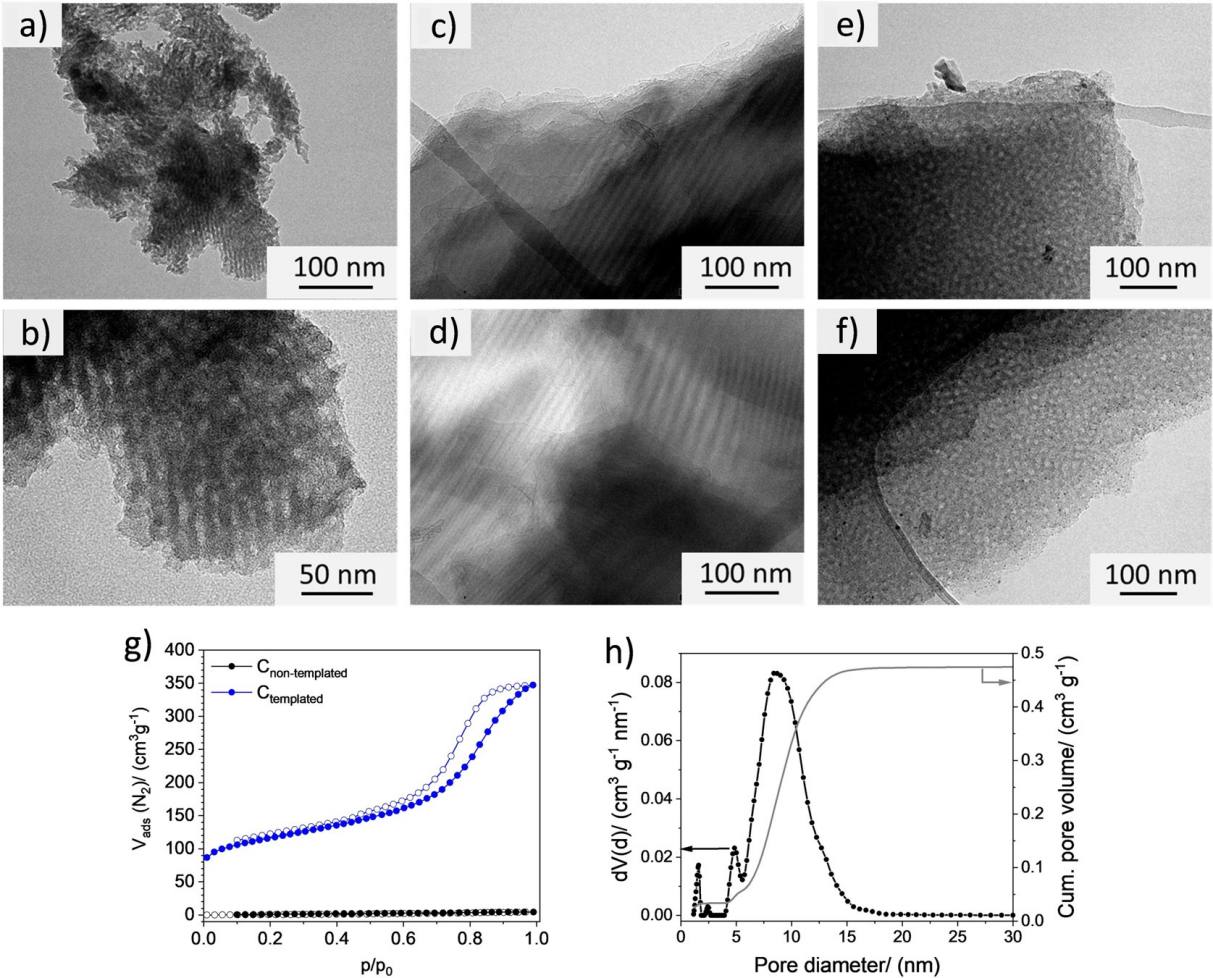
TEM Images of a), b) the templated carbon powder and c)–f) the carbon films. N_2_‐physisorption isotherms of the g) non‐templated (black) and templated (blue) carbon and h) the corresponding pore size distribution of the templated material measured at 77 K.

The pore size and surface properties of the material were estimated using carbon powders from the resol precursor (carbonized at 900 °C) via nitrogen physisorption measurements at 77 K. The non‐templated carbon has no appreciable SSA (Figure 2 g). With Pluronic F127 as surfactant a mesoporous carbon material with a SSA of 491 m^2^ g^−1^ and a pore volume of 0.5 cm^3^ g^−1^ was produced. The carbon shows a type IV(a) isotherm characteristic for mesoporous systems.^[40]^ The pore‐size distributions were calculated using quenched solid density functional theory for slit‐like pore geometry (Figure 2 g). With Pluronic F127, mainly mesopores with a size of around 10 nm were formed. The surfactant generated ordered micelles in the resol.^[38]^ The TEM images show an ordered hexagonal pore structure in the carbon powder (Figure 2 a). Using SAXS measurements the ordered pore structure also was confirmed (Supporting Information S.1.). In order to obtain more detailed information of the precursor characteristics at micro‐ and nanoscales, carbon thin films were formed by spin coating. The polymerized resin was either formed using temperature treatment (non‐templated) or by UV‐EISA (templated). The additional UV treatment supports the self‐assembly of surfactant and precursor molecules and enables a rapid hardening, required for the NIL‐process.

Templated thin films (≤2 μm, 900 °C) were characterized by TEM measurements after scraping off sections from the substrate (Figure 2 c–f) confirming the ordered hexagonal pore structure in the EISA‐generated films. Interestingly, within the films ordered pores with different structures or orientations occur, so regions with pores arranged vertically and horizontally are found. We conclude that the templating also works at microscale. The electrochemical properties of the carbon films were investigated with respect to carbonization temperatures varying from 700 to 1000 °C. The specific resistances are studied via four‐point measurements on carbon thin films (Table 1). Typically, electrical conductivity is expected to increase with increasing carbonization temperatures due to a higher graphitization, however in a film, macroscopic cracks may cause a growing resistance.^[41]^ We measured the lowest electrical resistance for the 900 °C carbonized sample for both carbon materials. At 1000 °C cracking in the film starts to affect the films. Overall the templated sample shows the lowest resistance with 1.2×10^−5^ Ωm the surfactant additionally stabilizes the precursor and prevents crystallization and cracking.

**Table 1:** Specific four‐point resistances of carbon thin films after different carbonization temperatures.

*T* [°C]	Specific resistance [Ω m]	
	Non‐templated	Templated
700	2.1×10^−2^	1.2×10^−2^
800	1.2×10^−4^	1.5×10^−4^
900	1.2×10^−4^	1.2×10^−5^
1000	–	2.5×10^−4^

### Nanoimprint lithography

The basic requirements for interdigitated electrodes are crack‐free electrode fingers which are perfectly separated from each other. Therefore the lines must have an adequate and homogeneous height to avoid dimension‐variation related resistance fluctuations. Furthermore, short spacings in between the fingers facilitate the ion transport.[^30^, ^42^] Finally, carbonization should lead to high surface area carbons with controlled pore size in the given structure on the substrate. The nanoimprint lithography is ideally suited to produce patterned surfaces at micro‐ and nanoscale.^[43]^ In the following, we show the printing of interdigitated micro‐structures with line widths down to 250 nm. The line widths and spacings of the structures are shown in Table 2.

**Table 2:** Geometrical parameters of the printed interdigitated structures.

Structure	Line width	Distance	Length
IDE250	250 nm	1 μm	400 μm
IDE500	500 nm	10 μm	400 μm

**Figure 3:**
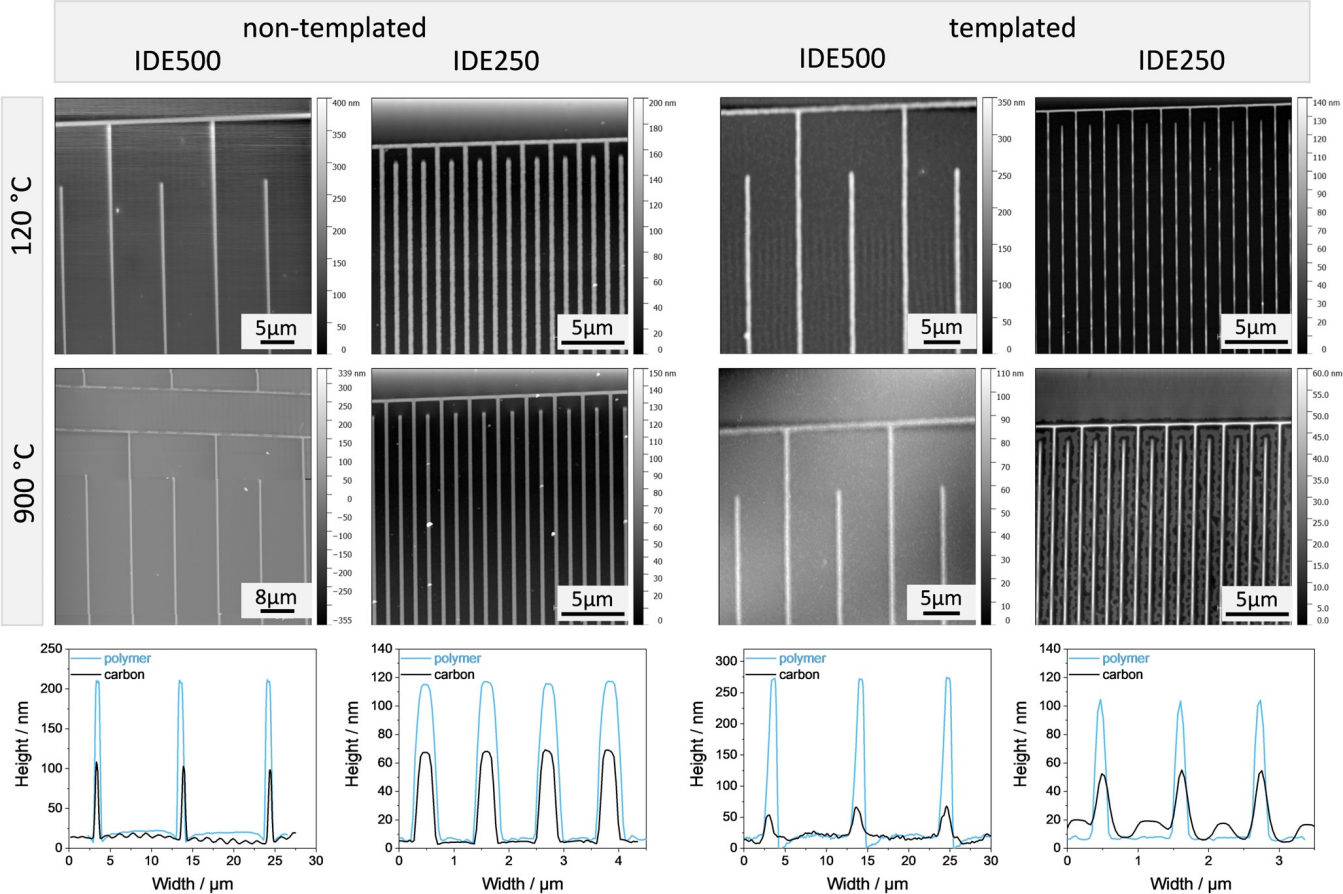
AFM images and height profiles (polymer blue lines, carbon black lines) of the different structures after printing and after pyrolysis.

The “green resol” precursor is excellent for the application in the SA‐NIL process because of its adjustable viscosity and the moderate curing conditions. The used solvent ethanol is convenient for the NIL process because of low evaporation temperatures and the inertness of the polydimethylsiloxane (PDMS) stamps against it. The ethanol easily diffuses through the membrane without inducing swelling of the PDMS. For the pure resin, a temperature treatment at 120 °C is necessary for the complete curing of the precursor during the NIL‐process. Using this precursor, homogeneous and defect‐free electrodes with negligible precursor residue in between the lines were printed (Figure 3). The used PDMS stamps have channels with a depth of 500 nm. The amount of solvent in the precursor is 84 wt/% which is completely removed during printing. This fact causes a massive shrinkage of the polymer and lines after the NIL process. After printing IDE500 remains with line heights of around 200 nm and IDE250 with 120 nm. After carbonization stable interdigitated structures were formed with typical line heights of 100 (IDE500) and 60 nm (IDE250), thus indicating a volume reduction of 50 %.

The surfactant in the precursor supports the formation of homogeneous and defect free lines. For the small interdigitated structure IDE250 the amount of Pluronic F127 was reduced in order to form completely separated electrodes without residual films in between. Possible residues shrink and vanish during pyrolysis and will not shortcut the electrodes. For IDE250 line heights up to 100 nm and for IDE500 250 nm were reached. Nevertheless, the NIL process was satisfying for this system. Interdigitated structures with completely contacted and homogeneous fingers were printed. Also, after pyrolysis at 900 °C the structures stayed defect‐free with moderate line heights for the envisioned electrochemical applications. The non‐templated IDE500 structures resulted in line heights of 100 nm. Compared to those, the templated IDE500 structures again showed a drastic shrinkage, because of the surfactant removal. The lines remained smaller than the non‐templated lines (50 nm). Due to the small carbon volume and mass it is difficult to investigate the porosity and the arrangement of pores in the interdigitated structures. The lines in IDE250 were between 30 and 50 nm in height. All generated patterns showed promising characteristics for further micro‐supercapacitor applications.

### Electrochemical characterization

The basic electrochemical characteristics of the carbon materials were investigated in thin film EDLCs. Therefor freestanding carbon films (thickness=2 μm) were attached with the PVA/H_2_SO_4_‐hydrogel electrolyte and a polypropylene separator. The non‐porous (non‐templated) carbon material reached a specific areal capacitance of 5.4 mF cm^−2^ (Supporting Information S.2.). Compared to that, the soft templated carbon film reached a slightly higher capacitance (6.0 mF cm^−2^). Depending on the orientation of the hexagonal pores the accessibility for the electrolyte ions varies. As a result the actual capacitance was smaller than expected based on SSA data.

**Figure 4:**
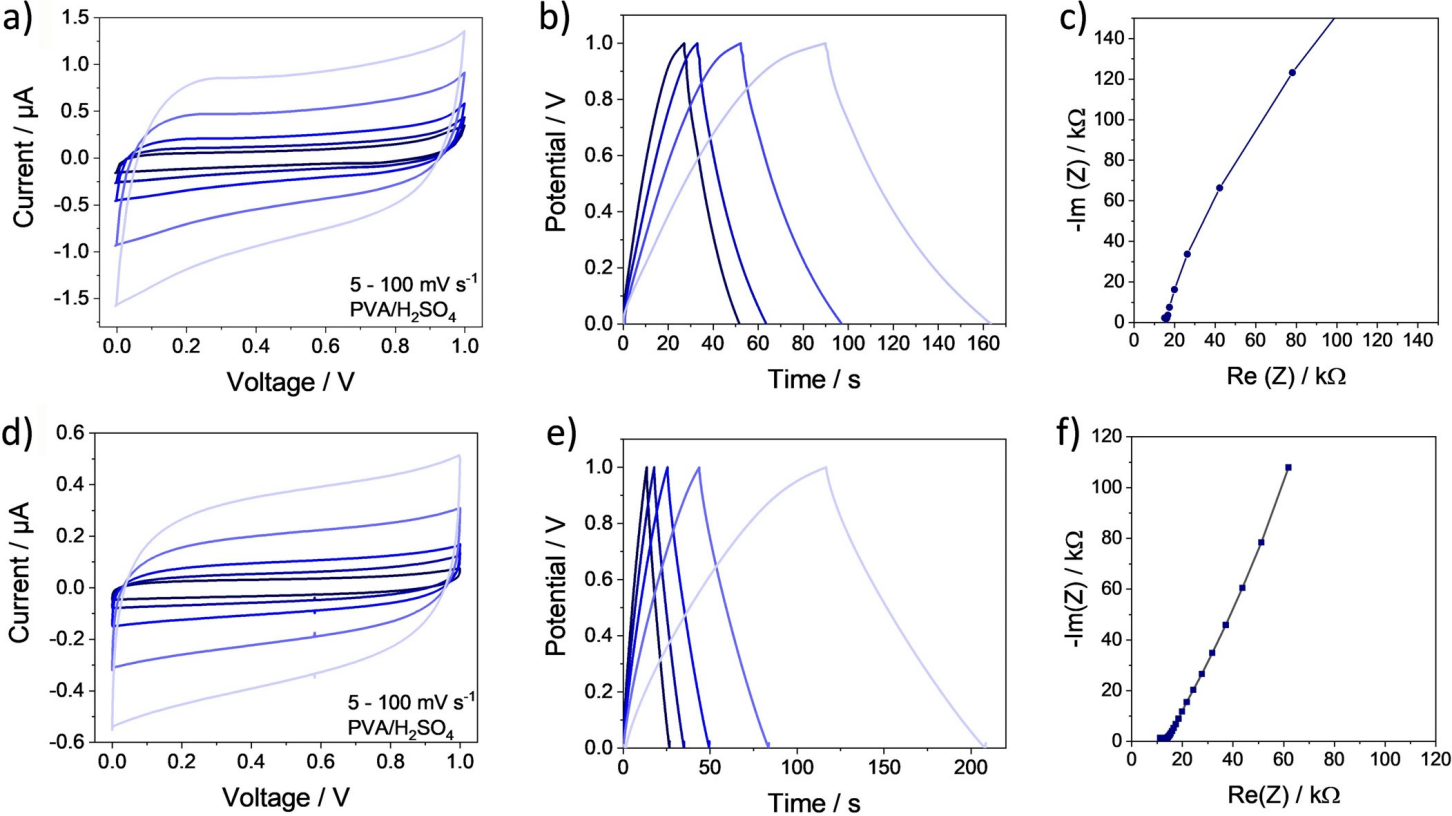
Electrochemical characterization of IDE500 (top) and IDE250 (bottom) by CV (left), GCD (center), and Nyquist plot (right) using the templated carbon material.

The carbonized interdigitated structures were firstly contacted with a thin gold layer at the outer contacts. After isolation with a PMMA ring the structures were activated in a cold Ar plasma in order to form hydrophilic surface groups. After that a thin PVA/H_2_SO_4_ film was attached. The electrochemical characterization by cyclic voltammetry, galvanostatic charge‐discharge (GCD) and impedance spectroscopy is shown in Figure 4 (Supporting Information S.3.). CV‐curves of the porous carbon micro‐supercapacitors show the typical rectangular shape even at higher scan rates. The calculated capacitances are listed in Table 3.

**Table 3:** Calculated device capacitances and the areal capacitance of the MSCs.

Structure	Precursor	Capacitance [mF cm^M‐>2^]	
		Device	Areal
IDE500	non‐templated	0.06	0.3
	templated	0.21	1.7
IDE250	non‐templated	0.02	0.2
	templated	0.03	0.8

The Nyquist plots of the MSCs display the expected shape for capacitive behavior (Figure 4 c). An equivalent series resistance (ESR) of around 15 kΩ was observed that was mainly influenced by the small line width in the interdigitated structure. Typically, the capacitance increases with decreasing electrode spacings.^[44]^ In our case we observed the reverse trend. For the IDE500 higher device capacitances were reached. A significant influence on the performance of the MSC originates from the line width relating to the electrode resistance. With smaller lines also the overall line height decreases and higher resistance values effectively reduce the performance. Smaller lines also increase the possibility of small cracks in the electrode fingers. Nevertheless also for the small IDE250 structure promising capacitances were reached.

**Figure 5:**
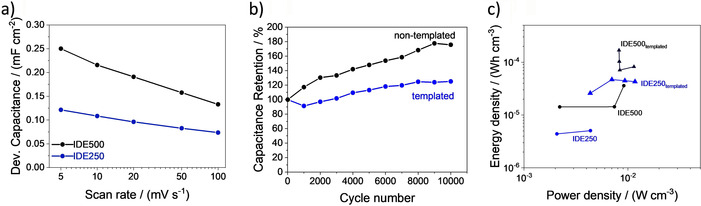
a) Rate capability for both templated structures, b) GCD cycling stability over 10 000 cycles for the template and non‐templated IDE250 and c) the Ragone plot for the non‐templated (•) and templated (▴) MSCs.

Furthermore a stark influence of the surfactant in the precursor is evident. Both structures showed a capacitance increase with additional soft templating, indicating, that even in these small structures the surface area can be enhanced. For the larger structure the increase was higher than for the smaller structures. In the IDE250 the higher probability of pore collapse is disadvantageous. Regarding shorter diffusion length the IDE250 had a significant advantage. The smaller structure shows a better rate capability than the IDE500 (Figure 5 a).

The cycling stability of the MSCs was shown exemplarily for the IDE250 structure. The MSC was charged and discharged over 10 000 cycles demonstrating an excellent cycling stability over the numerous cycles with a capacitance retention of 120 % for the template and 180 % for the non‐templated MSC. The slight increase originates from improved wetting by the hydrophilic electrolyte on the carbon surface over the first cycles.

The leakage current of these micro‐supercapacitors was approximately 10^−3^ μA for the templated and around 10^−4^ μA for the non‐templated samples (Supporting Information S.4.). As already mentioned, the surfactant also supports the formation of homogeneous structures. During the printing process the self‐assembly occurs within a few seconds. Therefore, residues could remain in between the electrode fingers which were not completely displaced by the stamp. These residual shunts cause the increased self‐discharge. In Figure 4 c energy and power density of the different MSCs were compared in a Ragone plot. In correlation with the calculated capacitances, higher energy densities were reached by the templated MSCs in comparison to the non‐templated ones. Also, the power densities were increased for the templated systems. The power density was influenced by two important electrode properties. Firstly, the serial electrode resistance was a crucial factor. As discussed before, larger lines decrease the resistance enabling higher power and current density. At the same time the length of the diffusion pathways is important. Faster diffusion also generates enhanced power capability. Based on these compensating effects the power densities of the templated structures cover a similar range. Corresponding to these measurements relaxation time constants were calculated (Supporting Information S.5.). The IDE500 shows the best relaxation time constants of 50 (non‐templated) and 70 ms (templated). In comparison with other solid‐state carbon based MSCs for the presented cells the capacitances and energy densities are in the same ranges (Supporting Information S.6.) despite of the much smaller lines and higher resolutions. In terms of power densities and charging times, the high electrode resistance caused by the small interdigitated fingers limits the performance. An additional heteroatom doping or geometry optimization could further improve the performance.

## Conclusion

A soft templated “green resol” based precursor system was successfully used as an ink in the SA‐NIL‐process. High resolution interdigitated patterns down to 250 nm were printed and transferred into stable highly porous carbon electrodes. For the first time, mesoporous carbon electrodes were processed at such tiny line width for a quasi‐solid‐state micro‐supercapacitor using PVA/H_2_SO_4_‐hydrogel electrolytes. The devices show excellent supercapacitor characteristics with cycling stabilities over 10 000 cycles.

## Experimental Section

**Carbon precursors**: The precursors were synthesized using 82 mg (0,65 mmol) phloroglucinol (99 %, Merck) and 61 mg (0,82 mmol) glyoxylic acid (97 %, Alfa Aesar) dissolved in 1 mL ethanol (99 %, VWR). For the soft templating 161 mg (97 mg for IDE250) Pluronic F127 (Sigma–Aldrich) were added to the solution.

**Compact film formation and nanoimprint lithography**: Thin films and structures were prepared on pre‐treated boro‐aluminum silicate slides (Corning 1737, DELTATechnologies) (25 mm×25 mm×1.1 mm). The pre‐treatment for surface activation was performed in a piranha solution (1 part 30 % H_2_O_2_, 3 parts conc. H_2_SO_4_) for at least 20 min. Thin films were spin coated (Spin 150, ATP GmbH) using 80 μL of precursor solution with a speed of 2000 rpm over 30 s. The films were dried at 120 °C. Soft templated thin films additionally were treated for 50 min in a UV box (Hoenle, 20 mW cm^−1^).

The solvent assisted nanoimprint lithography was performed with the Micro‐Contact‐Printing System μ‐CP 3.0 from GeSiM mbH and patterned PDMS stamps (Sylgard 184 elastomer kit, Dow Chemicals). The process of stamp preparation and molding is published elsewhere.^[45]^ For the printing process a droplet (4 μL) of the precursor solution was deposited on the substrate and later the stamp was pressed into it. Thereby the solution was displaced into the spaces of the structured stamp. The substrate was then heated to 120 °C for 15 min in order to induce the polymerization and evaporate the solvent. The precursor containing surfactant was treated at 80 °C for 30 min with an additional exposure of UV light (Delolux‐04, Delo, 8 mW cm^−2^) through the stamp membrane. After treatment the stamp was peeled off and the precursor structure remains. Interdigitated structures with line width of 500 (IDE500) and 250 nm (IDE250) were printed.

TEM measurements were carried out using a JEM 1400plus Microscope (120 kV). The samples were applied on a copper grid using a suspension of the carbon material in acetone. For the measurement of thin films, they were scraped off from the substrate. AFM measurements were performed via a Dimension 3000 (Digital Instruments) in tapping mode. Small‐angle X‐ray diffraction (SAXS) experiments were carried out on a Bruker Nanostar diffractometer in transmission mode with Cu_Kα1_ radiation (0.15405 nm) coupled with a position sensitive HiStar detector. N_2_‐Physisorption measurements were carried out in a Quadrasorp EVO/SI (3P instruments) at 77 K. Previously the samples were degassed at 150 °C for 12 h. The SSAs of the materials were calculated using the multipoint BET method. The pore size distributions were calculated assuming slit‐like pore geometry using quenched solid density functional theory (QSDFT) method.

**Carbonization**: Carbon materials were produced in three different ways: 1) Carbon films and 2) carbon interdigitated structures on substrates, furthermore, 3) carbon powders were formed for further characterization. All carbonizations were carried out at 900 °C for 2 h with a heating rate of 150 K h^−1^ under argon. For powder synthesis the precursor was hardened by treatment with UV for 20 min.

**Preparation of micro‐supercapacitors**: A polymer hydrogel based on polyvinyl alcohol (PVA; Merck; molecular weight 145 000) and sulfuric acid was used as electrolyte. Therefor 0.5 g of PVA was dissolved in 7 mL of deionized water at 90 °C with vigorous stirring. In the next step 0.5 g of conc. sulfuric acid was added to the solution. With that, the electrolyte was ready for coating and drying at room temperature.

For the preparation of the symmetric full cell setup, free‐standing carbon films with a thickness about 1 μm were produced. The films can be detached from the substrates by soaking in acetone. After drying at 70 °C the films were placed on two titanium pistons (12 mm diameter) with electro‐DAG. As for the structured micro‐supercapacitors the carbon surface was activated with Ar plasma and the hydrogel electrolyte was deposited on the electrodes. The electrodes were assembled with a polypropylene separator in a Swagelok setup.

The carbonized interdigitated structures were cleaned from extra carbon residuals. The electrode area was masked with silicone during the deposition of a chrome (10 nm) and gold (100 nm) current collector by physical vapor deposition (B39, Malz & Schmidt) with a deposition rate around 15–20 kÅ s^−1^. After that, the mask was removed and a ring of PMMA was deposited around the interdigitated area as an isolation of the current collector. Moreover, the ring serves as a reservoir for the electrolyte. Finally, the electrode surface was activated using an Ar plasma treatment (KINpen, neoplas tools) in order to generate a more hydrophilic surface and 10 μL of the hydrogel electrolyte were added. After evaporating water excess at room temperature, the quasi‐solid‐state electrolyte remains.

**Electrochemical testing**: All cells were tested in a VMP3 Potentiostat from Bio‐Logic and cyclic voltammetry (CV) electrical impedance spectroscopy (EIS) and galvanostatic cycling were performed. CVs were recorded with the hydrogel electrolyte PVA/H_2_SO_4_ as electrolyte between 5 and 100 mV s^−1^ in a potential range between ±1 V. EIS was carried out in a frequency range between 5 mHz and 100 kHz. Galvanostatic cycling was executed in the voltage range of 0–1 V at a current density of 0.6–2.5 mA cm^−2^.

The specific capacitances are calculated using CV data and GCD‐data. For the capacitance calculation out of CV‐data, the area of the charge‐ and discharge curve is integrated and normalized to the voltage window Δ*U* and the scan rate *υ* [Eq. 1]:

(1) $$C=\frac{\int_{0}^{1}I\;dU}{\Delta U\;v\;}$$

The GCD capacitance is calculated using the discharge curve and the charging current [Eq. 2]:

(2) $$C=2I{\left(\frac{dU}{dt_{diss}}\right)}^{-1}$$

For normalization the device area and device volume (including electrolyte volume and area) are used. Furthermore, an areal capacitance based on the effective carbon electrode area is calculated.

Energy and power density are calculated by integration of the galvanostatic discharge curves using Equations. 3 and 4:

(3) $$\;E_{vol.}=\frac{I\;•\;\int U\;dt_{Dis}}{V_{D}•3600\;}$$

(4) $$P_{vol.}=\frac{E_{vol.}}{t_{Dis}\;}$$

The area of the integrated discharge curve *A*_Dis_, the applied current *I*, *t*_diss_ discharge time and the device volume of interdigitated electrodes *V*_D_ are used as factors for the calculation.

## Conflict of interest

The authors declare no conflict of interests.

## Supporting information

As a service to our authors and readers, this journal provides supporting information supplied by the authors. Such materials are peer reviewed and may be re‐organized for online delivery, but are not copy‐edited or typeset. Technical support issues arising from supporting information (other than missing files) should be addressed to the authors.

Supplementary
