# Supplementary material for: Green Precursors and Soft Templating for Printing Porous Carbon‐Based Micro‐supercapacitors
Source: Chemistry. 2020 Dec 7;27(4):1356–63. doi: 10.1002/chem.202003124 (PMC7898350; doi:10.1002/chem.202003124)
Supplement: Supplementary file 1 — Supplementary [file CHEM-27-1356-s001.pdf]

Chemistry–A European Journal

Supporting Information

Green Precursors and Soft Templating for Printing Porous Carbon-Based Micro-supercapacitors

Stefanie Lochmann, Susann Kintzel, Yannik Bräuniger, Thomas Otto, En Zhang, Julia Grothe, and Stefan Kaskel*[a]

Supporting Information

S.1. SAXS measurements

SAXS measurements confirm the ordered pore structure. The precursor system was optimized for the NIL and with this the ratios of solvent, surfactant and starting material differ from the optimized ratios needed for the formation of highly ordered pore systems. Besides this, the reflexes indicating the porous structure are obvious.

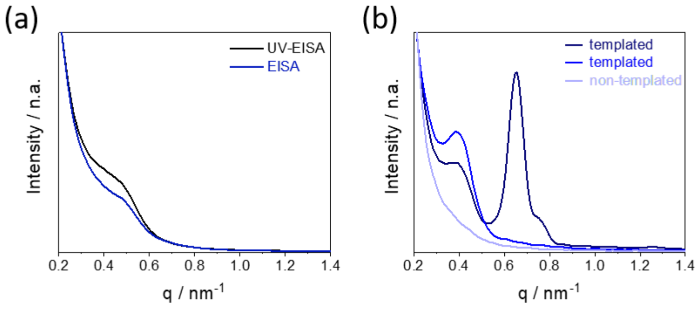

Figure S 1: SAXS-Pattern of the carbon powder material after EISA and UV-EISA process (a) and of carbon thin films (b).

S.2. Thin film EDLCs

For comparison symmetric thin film EDLCs were measured for the different materials:

	Areal capacitance [mF cm^{-2}]	Vol. capacitance [F cm^{-3}]
Non-templated resol	5.4	66.0
templated resol	6.0	79.6

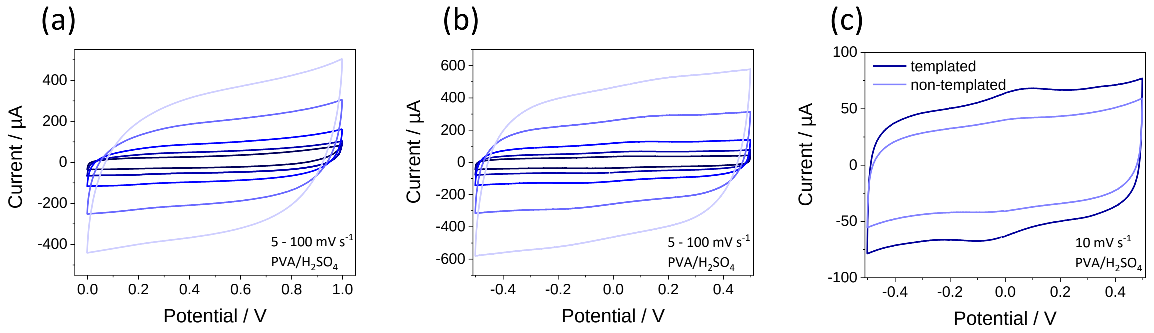

Figure S 2: CV-curves of the thin film EDLCs: (a) non-templated, (b) templated and (c) comparison at 10 mV s^{-1}.

S.3. Electrochemical data of non-templated MSCs

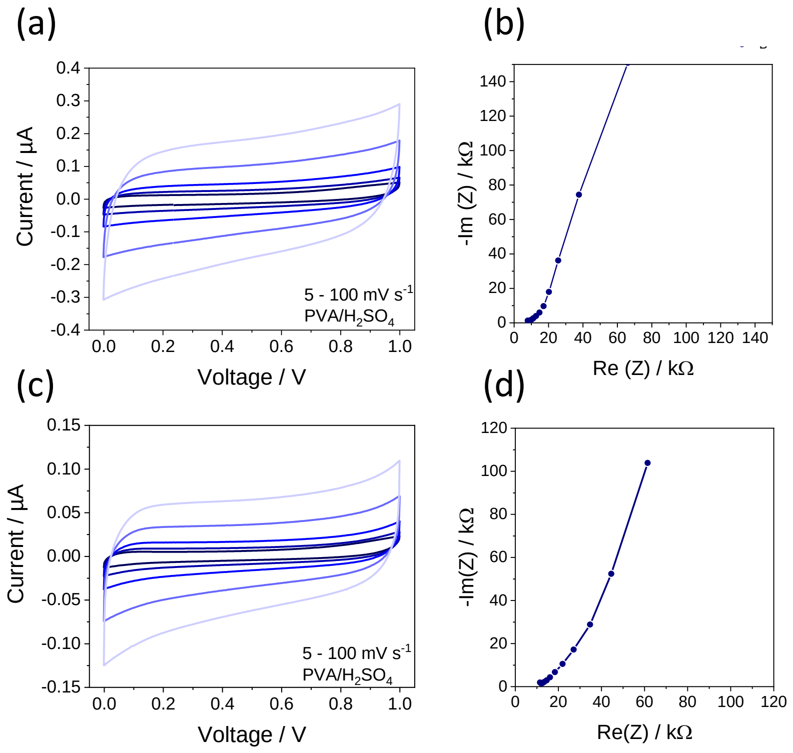

Figure S 3: CV-curves and Nyquist-plots of the non-templated MSC (IDE500 (a,b) and IDE250 (c,d)).

S.4. Leakage current characteristics

Leakage current is calculated measuring the self-discharge in open circuit after pre-charging the micro-EDLC to 1 V. The corresponding leakage current was estimated using the following equation:

$$I_{leak} = C \cdot \frac{dV}{dt}$$

Where I_{leak} is the leakage current, C is the capacitance obtained from the CV curves dV/dt is the discharge rate.

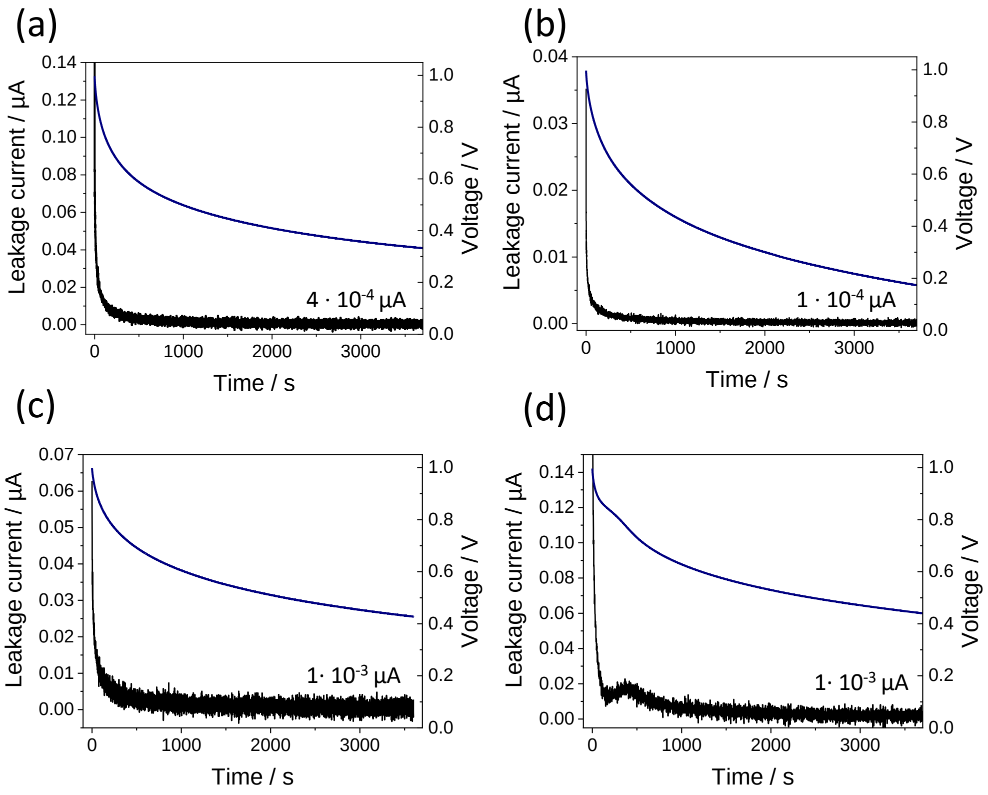

Figure S 4: Leakage current and the corresponding voltage of the different MSCs: non-templated IDE500 (a), IDE250 (b), templated IDE500 (c) and IDE250 (d).

S.5. Relaxation Time Constants

Out of impedance spectroscopy data the relaxation time constants are calculated.

Table S 1: Relaxation time constants of the different materials and strucutres.

Structure	Material	Time constant τ_0 / s
500/10	non-templated	0.05
250/1	non-templated	0.43
500/10	templated	0.07
250/1	templated	0.52

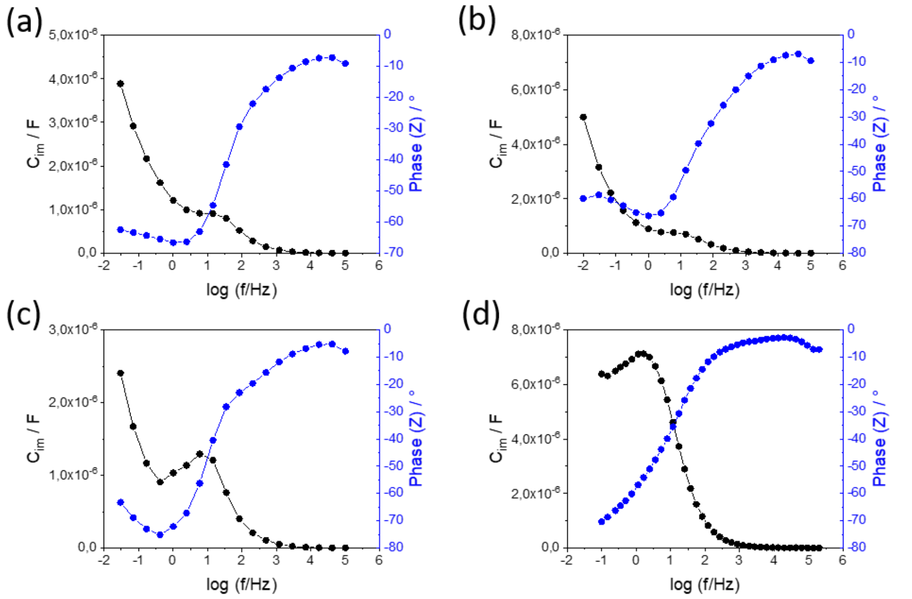

Figure S 5: Imaginary capacitance and phase angle plotted against the frequence for the non-templated IDE500 (a) and IDE250 (b) and the templated MSC with IDE500 (c) and IDE250 (d).

S.6. Classification

Method	Material	Electrolyte	Capacitance	Energy density	Power density	Ref.
laser structuring	porous carbon	PVA/H_2SO_4	9 mF cm^{-2}	1.8 mWh cm^{-3}	0.7 W cm^{-3}	[1]
3D printing	CNTs	PVA/H_3PO_4	5 mF cm^{-2}	0.1 mWh cm^{-3}	3.7 W cm^{-3}	[2]
photo-lithography	rGO	PVA/H_2SO_4	0.1 mF cm^{-2}	3.6 mWh cm^{-3}	1270 W cm^{-3}	[3]
plasma etching	CNTs	PVA/H_3PO_4	2 F cm^{-3}	0.1 mWh cm^{-3}	30.4 W cm^{-3}	[4]
Inkjet printing	graphene	PVA/H_2SO_4	0.7 mF cm^{-2}	1.0 mWh cm^{-3}	0.1 W cm^{-3}	[5]
NIL IDE250	templated resol	PVA/H_2SO_4	0.6 mF cm^{-2}	0.02 mWh cm^{-3}	4 mW cm^{-3}	
NIL IDE500	templated resol	PVA/H_2SO_4	2.0 mF cm^{-2}	0.2 mWh cm^{-3}	9 mW cm^{-3}	

Literature

[1] P. Yadav, A. Basu, A. Suryawanshi, O. Game, S. Ogale, *Adv. Mater. Interfaces* **2016**, *3*, 1–9.

[2] W. Yu, H. Zhou, B. Q. Li, S. Ding, *ACS Appl. Mater. Interfaces* **2017**, *9*, 4597–4604.

[3] Z. S. Wu, K. Parvez, X. Feng, K. Müllen, *J. Mater. Chem. A* **2014**, *2*, 8288–8293.

[4] L. Liu, D. Ye, Y. Yu, L. Liu, Y. Wu, *Carbon N. Y.* **2017**, *111*, 121–127.

[5] J. Li, S. Sollami Delekta, P. Zhang, S. Yang, M. R. Lohe, X. Zhuang, X. Feng, M. Östling, *ACS Nano* **2017**, *11*, 8249–8256.
